# Photo-/Electrocatalytic Reduction of CO_2_ Based on Ferroelectrics

**DOI:** 10.34133/research.0843

**Published:** 2025-08-14

**Authors:** Rui Wei, Shun Li, Andy Li, Yuan-Hua Lin

**Affiliations:** ^1^ State Key Laboratory of New Ceramics and Fine Processing, School of Materials Science and Engineering, Tsinghua University, Beijing 100084, China.; ^2^ School of Chemistry and Chemical Engineering, Jiangsu University, Zhenjiang 212013, China.

## Abstract

Converting CO_2_ into sustainable fuels or useful carbon-derived substances offers an effective strategy for tackling the energy crisis and combating global warming. Photo-/electrocatalytic CO_2_ reduction provides a simple, energy-efficient, and environmentally friendly route compared to existing technologies. Ferroelectric materials, characterized by their unique spontaneous and switchable polarization, have emerged as promising candidate catalysts due to their controllable surface physical and chemical properties. This review commences by summarizing the mechanism underlying ferroelectric polarization-modulated photo-/electrocatalytic CO_2_ reduction. We then highlight the recent key advancements in CO_2_ reduction utilizing ferroelectric materials, along with their performance promotion strategies. Finally, we explore the obstacles and future perspectives in this field. This review will provide useful guidance for systematic design and advancement of effective photo-/electrocatalysts based on ferroelectric materials.

## Introduction

Global climate change and the energy crisis pose challenges to the modern society. Carbon dioxide (CO_2_), generated from industrial and other human activities, is the primary greenhouse gas in the atmosphere. Its emissions reached 41.6 Gt/year in 2024 (Global Carbon Budget, 2024), with its concentration directly correlated to rising global temperatures [1–4]. The escalating greenhouse effect threatens ecological sustainability and human development. Consequently, transforming CO_2_ into value-added chemicals and fuels can not only effectively mitigate greenhouse gas emissions but also provide a sustainable solution for future energy demands [5,6]. In this context, developing efficient CO_2_ conversion technology has emerged as a long-term solution for the circular economy and a key focus of research in materials science and chemical engineering [7–9].

In recent years, electrocatalysis and photocatalysis CO_2_ reduction (CO_2_RR) have demonstrated significant potential for transforming CO_2_ into valuable chemicals or fuels under mild conditions [10–15]. These approaches not only contribute to the reduction of greenhouse gas but also facilitate the realization of a sustainable carbon cycle [16]. Consequently, these straterties have garnered substantial attention from researchers across a wide range of disciplines. However, great challenges hinder their practical applications. Existing electrocatalytic technologies (e.g., those using Cu-based catalysts [17]) often exhibit a Faraday efficiency of less than 30% for C_2+_ products, with overpotentials typically exceeding 450 mV. Furthermore, the process generates a substantial number of by-products, thereby posing a considerable challenge to the selective production of specific target products [18]. Additionally, it is crucial to minimize the high overpotential encountered during electrocatalysis and reduce the cost of electrocatalysts, which typically contain noble metals [19]. As for the photocatalytic process, it is confronted with multiple challenges, including a narrow range of light absorption and rapid recombination of photogenerated electron–hole pairs, which significantly impair efficiency and selectivity of CO_2_RR [13,20,21].

Ferroelectric materials exhibit unique spontaneous polarization characteristics that can be reversed by an external electric field [22], making them promising for applications in sensors, capacitors, microdrivers, and other fields [23]. Recently, the potential of ferroelectric materials in improving the efficiency and selectivity of electrocatalytic and photocatalytic reactions has gained attention. Unlike conventional catalysts, which are constrained by static surfaces that hinder dynamic regulation of catalytic pathways, ferroelectric polarization-induced internal electric field in ferroelectric materials can modulate carrier transport and surface states, thereby enhancing electrocatalytic and photocatalytic performance [24]. Specifically, the internal electric field provides a strong driving force to promote electron–hole separation and migration toward opposite directions. Furthermore, the switching of spontaneous electric polarization can alter the reactivity of the catalyst’s surface, allowing for tunable adsorption and binding strengths of reaction intermediates [25]. These intriguing effects make ferroelectric materials increasingly attractive in the field of photo-/electrocatalysis, especially regarding CO_2_ activation and conversion [26,27].

Here, we provide a comprehensive review of the emerging field of photo-/electrochemical reduction of CO_2_ using ferroelectric materials, mainly focusing on mechanistic insights and material design strategies. We begin by outlining the fundamental principles of CO_2_RR and ferroelectric materials/polarization. Next, we discuss how ferroelectric polarization enhances electrocatalytic and photocatalytic CO_2_ conversion by modulating the electronic structure, surface characteristics of catalyst, and charge stransfer behavior. Subsequently, we present the state-of-the-art representative ferroelectric photo-/electrocatalysts for CO_2_RR and summarize the latest advancements in performance-enhancement strategies. This review paper aims to offer an in-depth investigation of ferroelectric polarization-driven photo-/electrocatalysis, providing a new perspective on optimizing CO_2_ conversion technology and advancing sustainable energy solutions.

## Mechanisms of Photo-/Electrocatalytic CO_2_RR Using Ferroelectrics

### Ferroelectrics and ferroelectric polarization

**Fig. 1.**
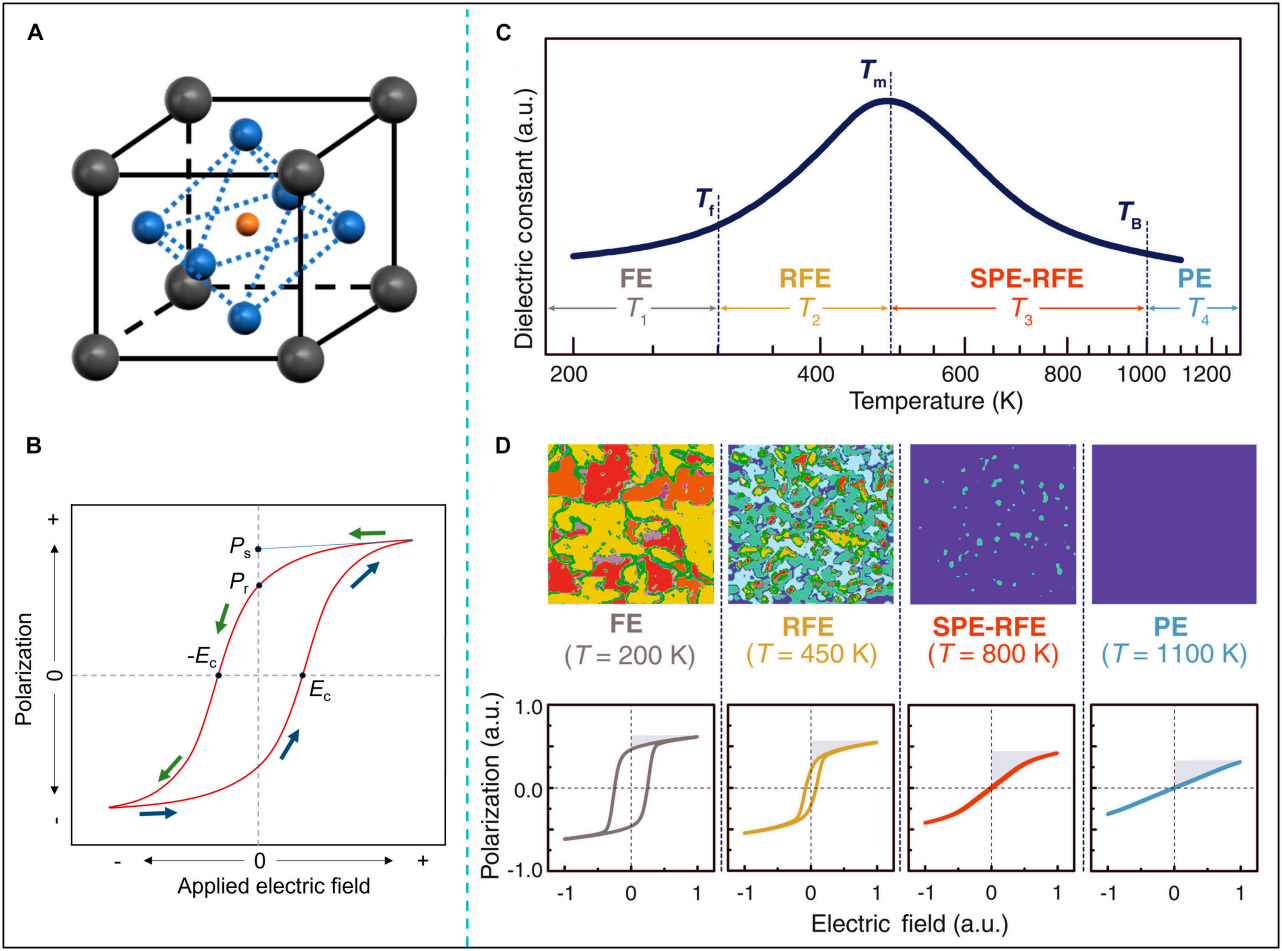
Schematic illustration of (A) perovskite structure and (B) ferroelectric hysteresis loop. (C) Simulated temperature-dependent dielectric constants for Sm-doped BiFeO_3_-BaTiO_3_ (Sm-BFBT). *T*_1_ to *T*_4_ are temperature bands according to the characteristic *T*_f_, *T*_m_, and *T*_B_ values. (D) Simulated domain configurations and corresponding polarization–electric field (*P*–*E*) hysteresis curves demonstrate distinct characteristics of ferroelectric (FE), relaxor ferroelectric (RFE), superparaelectric RFE, and paraelectric (PE) phases. The nonpolar phase is depicted in blue, while multicolored regions correspond to domains with differing polarization orientations. Energy density distributions are represented by shaded regions within the *P–E* loops. Reproduced with permission from [140]. Copyright 2021 AAAS.

Ferroelectrics are a category of materials characterized by spontaneous polarization, first documented by Valasek in 1921 in Rhosi salt [28]. Common ferroelectrics include perovskites (e.g., BaTiO_3_, BiFeO_3_, and Na_1-*x*_K*_x_*NbO_3_) (Fig. 1A) [29–31], Bi-based layered structures [32], potassium dihydrogen phosphate (KH_2_PO_4_), tungsten bronze (e.g., Ca_0.28_Ba_0.72_Nb_2_O_6_), fluorate [e.g., polyvinylidene difluoride (PVDF)] [33], and ilmenite (e.g., LiNbO_3_ and LiTaO_3_) [34]. The spontaneous polarization of ferroelectrics arises from the inherent asymmetry in their crystal structure, resulting in a mismatch between positive and negative charge centers and creating an electric dipole moment [35]. When an electric field is applied, the electric dipole moments within the material are rearranged, causing the polarization direction to align with the applied electric field. This direction of polarization can be altered in response to variations in the intensity of the applied electric field.

A critical phase transition temperature exists between the polarized and nonpolarized phases of ferroelectrics, which is referred to as the Curie temperature (*T*_c_). Below *T*_c_, ferroelectrics exhibit spontaneous polarization and ferroelectric properties. Above *T*_c_, ferroelectrics undergoes a phase transition into a nonpolarized phase, displaying paraelectric behavior. In contrast to conventional ferroelectrics, relaxation ferroelectrics do not possess a distinct Curie temperature. Instead, their characteristic temperature is defined as the temperature at which the maximum dielectric constant (*T*_m_) is observed. Relaxation ferroelectrics exhibit ferroelectric phases below the freezing temperatures (*T*_f_) and paraelectric phases above the Burns temperatures (*T*_B_) (Fig. 1C and D). On the contrary, ferroelectric materials exhibit a characteristic nonlinear response to external electric fields, manifesting as hysteresis in the polarization–electric field (*P*–*E*) relationship (Fig. 1B). Remarkably, these materials preserve residual polarization even after field removal, demonstrating stable dipole moment retention. This unique remanent polarization property renders them particularly suitable for nonvolatile memory devices and energy-harvesting systems [36].

### Mechanisms of ferroelectric polarization-modulated photo-/electrocatalytic CO_2_RR

The mechanisms of photocatalytic and electrocatalytic CO_2_ reduction are complex processes involving multiple steps and reaction pathways [10,37,38]. These processes aim to efficiently convert CO_2_ into valuable chemicals or fuels, such as carbon monoxide (CO), methane (CH_4_), formic acid (HCOOH), and methanol (CH_3_OH), utilizing either light or electrical energy as the driving force. Understanding the reaction mechanism is key to modulating reaction selectivity and improving production yields.

**Fig. 2.**
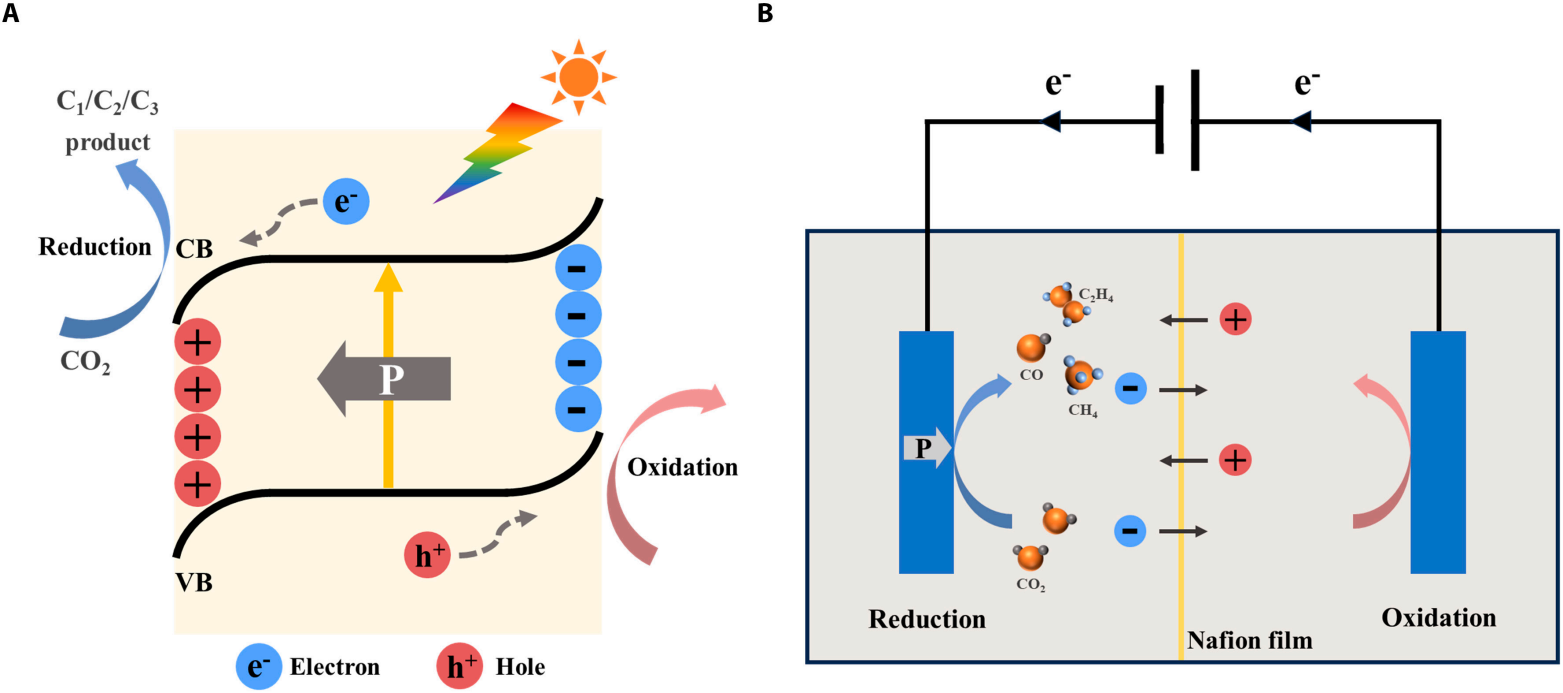
Schematic illustration of ferroelectric polarization-modulated (A) photocatalytic and (B) electrocatalytic CO_2_ reduction pathways.

In the photocatalytic process, as illustrated in Fig. 2A, semiconductor materials absorb photons, leading to the excitation of electrons from the valence band (VB) to the conduction band (CB), creating holes in the VB. These excited electrons then migrate to the catalyst’s surface to participate in the reduction of CO_2_, while the holes facilitate the oxidation of water to produce oxygen. Initially, CO_2_ molecules are adsorbed onto the catalyst’s surface, forming reactive intermediates such as HCOOH and CO. These intermediates can be further reduced through a variety of mechanisms, including coupled electron and proton transfer, to yield final products such as CH_3_OH and CH_4_ [39,40].

The electrocatalytic reaction is driven by electrical energy [41]. The mechanism can be illustrated in Fig. 2B. The reaction is typically carried out in an electrochemical cell divided into 2 parts with a Nafion film to prevent the product from being oxidized. During the electrocatalytic reaction, CO_2_ is adsorbed on the surface of the electrode (commonly used electrode materials include metals such as copper, cobalt, silver, and their compounds). Electrons provided by the applied voltage are transferred to the electrode surface, where they engage in the reduction reaction of CO_2_. At the same time, protons from the electrolyte are transferred to the electrode surface and combine with the electrons to form reactive intermediates. In electrocatalytic reactions, different electrode materials and reaction conditions can affect product selectivity. For example, copper-based catalysts typically produce C_2+_ products (e.g., ethanol and ethylene) [42], while silver-based catalysts primarily produce CO. Modulating the electronic structure, surface properties of the electrode materials and the reaction conditions can optimize the product selectivity and catalytic efficiency [9,43,44].

In 2004, Grosso et al. [45] first proposed the use of ferroelectrics as photocatalysts, opening up a new direction for advancing the research area of catalysis. Ferroelectric materials exhibit spontaneous polarization that generates an intrinsic electric field, which can be modulated through external thermal and electrical stimuli [46]. This built-in electric field promotes directional separation of photogenerated charge carriers (Fig. 2A), suppressing recombination losses and thereby boosting photocatalytic efficiency [24,47–49]. In the context of electrocatalysis (Fig. 2B), particularly in redox reactions involving electron gain and loss [50], the ability to absorb gases and transfer charges of the catalysts are crucial factors influencing the catalytic performance [51]. Thus, the charge-polarized surface and intrinsic electric field of ferroelectric materials critically enhance electrocatalytic performance through dual mechanisms: (i) efficient reactant activation and (ii) promoted charge carrier separation/transport, significantly boosting overall catalytic activity [52].

**Fig. 3.**
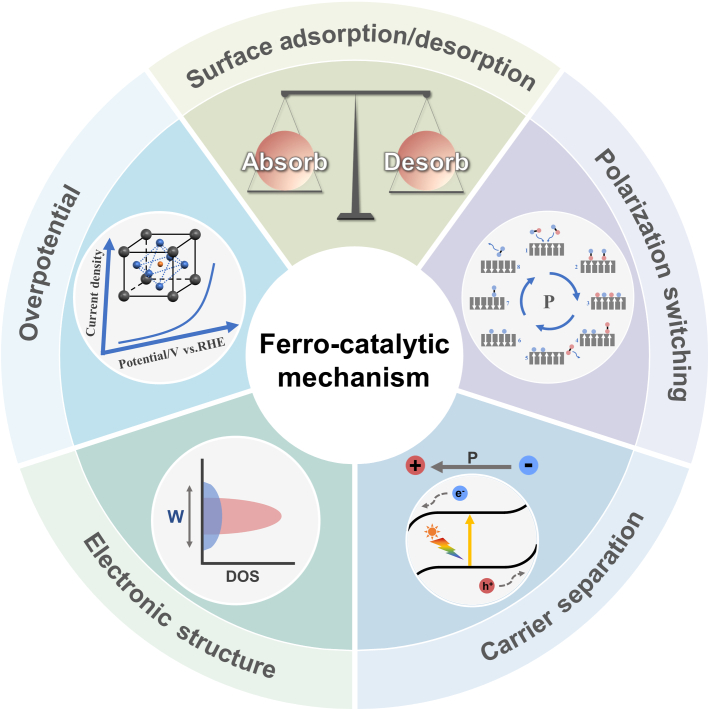
Schematic diagram of ferroelectric polarization-modulated catalytic mechanisms.

The unique spontaneous polarization and polarization reversal characteristics of ferroelectric materials provide a novel strategy to enhance the photo-/electrocatalytic performance of CO_2_RR. The tunable catalytic activity of ferroelectric catalysts mainly results from the built-in electric field and polarization effects inherent to the materials [53]. As illustrated in Fig. 3, these effects significantly affect the surface electron density/electronic structure, adsorption states of reactants and products, generation and separation of electron–hole pairs, and the transport behavior of charges, leading to changes in the catalytic reaction pathways and promoted selectivity and efficiency. The individual or synergistic effects of various factors collectively determine the product distribution and selectivity of CO_2_RR. The following sections will discuss how these mechanisms influence the CO_2_RR catalytic activity of ferroelectrics in detail [54].

#### Modulating the surface adsorption/reaction properties

Ferroelectric materials are characterized by spontaneous polarization, allowing the formation of a permanent electric dipole within the material. This creates a stable charge on the material’s surface. The built-in electric field influences the distribution of surface charges, leading to an increase in adsorption capacity of reactant molecules (e.g., CO_2_ and O_2_) and promotion of their activation [55]. In addition, the Sabatier principle dictates that optimal catalytic efficiency depends on appropriate interactions between the catalyst’s active site and the reactants [56]. Catalytic activity declines when reactant–catalyst interactions become either excessively strong or weak, highlighting the need for balanced material design. Ferroelectric systems address this challenge through polarization-state modulation, enabling precise tuning of surface chemical environments and adsorbate–catalyst interactions [57]. The polarization-switching ability of ferroelectric materials can reverse the electric field within the ferroelectric material to overcome the Sabatier limitation [58,59]. As a result, the reactant activation, surface reactivity, and reaction selectivity of the catalytic reaction can be regulated by controlling the polarization states of the ferroelectric material’s surface [60], offering a novel strategy for the rational design of catalysts.

#### Tuning the electronic structure

The electric polarization effect of ferroelectric material can significantly influence their electronic structure. The modulation of electronic states leads to the optimization of the reaction pathway and allows the catalyst to interact with the reactants in a more efficient manner, ultimately enhancing its catalytic performance. As a representative example, BiCoO_3_/Bi_5_CoTi_3_O_15_ composite catalysts illustrate how ferroelectric substrates enhance both the adsorption capacity and activation energy of the catalyst for specific molecules by providing additional electronic states [61].

In addition, it is well known that the alteration of the *d*-band center energy is closely associated with the adsorption energy of the reactants, which can significantly influence both catalytic activity and selectivity [62,63]. In ferroelectric materials, the *d*-band center may shift to different energy states as a consequence of the electric fields, which in turn influences the interaction forces and modes between the metal and the reactants [64]. As a result, the regulation of electron density on ferroelectric catalyst surfaces can effectively promote the adsorption of specific intermediates involved in catalytic reactions.

#### Promoting the carrier separation efficiency

Photocatalytic processes initiate through light-induced generation of electron–hole pairs in the semiconductor material [65]. Ferroelectric polarization establishes an internal electric field that induces band bending, thereby significantly suppressing charge recombination, facilitating efficient charge separation, and increasing the population of available charge carriers for surface redox reactions [66,67]. Consequently, the inherent polarization properties of ferroelectric materials can substantially enhance the efficiency of both photocatalytic and electrocatalytic CO_2_ reduction processes.

#### Lowering the overpotential of electrocatalysis

Ferroelectric materials have been reported to effectively reduce electrocatalytic overpotentials [68], including hydrogen evolution reaction (HER) [69,70], oxygen evolution reaction (OER) [52,71], and nitrogen reduction reaction (NRR) [72]. The built-in polarization electric field significantly accelerates the adsorption and activation of reactants by intensifying the electric field at the electrode–electrolyte interface [52]. This mechanism can lower the energy barrier for electron transfer, allowing ferroelectric materials to achieve higher reaction rates at lower applied voltages, thereby enhancing the overall catalytic performance of the system. A very recent theoretical study on Mo-BN@In_2_Se_3_ demonstrated an electrocatalytic pathway that is contingent upon polarization flipping [73]. The limiting value of the electrocatalytic no-reducing potential on Mo-BN@P↑-In_2_Se_3_ is 0.09 eV, which is well below the isolated Mo-BN monolayer (0.35 V). Mo-BN@P↓-In_2_Se_3_ is 0.53 eV, which highlights the distinctive capacity of ferroelectric materials to lower the overpotential through reversible polarization transitions while impeding competing reactions such as HER through a built-in electric field.

Moreover, the formation of double layer reduces activation energy and facilitates electron transfer [74]. At the electrode surface, the rearrangement of ions under an electric field leads to the formation of a charge-rich double layer, thereby optimizing the interactions between the reactants and the electrode surface. This double-layer effect is particularly significant in multi-electron conversion reactions, such as CO_2_RR, as it not only optimizes the adsorption modes of the reactants but also reduces the energy consumption of the multi-step reactions [75,76].

#### Polarization-dependent CO₂RR pathways

The ferroelectric polarization offers precise control of the CO_2_RR pathways by dynamically tuning the surface charge distribution and intermediate adsorption configuration. For instance, density functional theory (DFT) calculations reveal the critical role of polarization direction on the reaction energy: The upward polarization (P↑) in 2-dimensional (2D) ferroelectric In_2_Se_3_ is more favorable for driving the CO_2_ activation reaction: *CO_2_→*OCOH, which ultimately tends to produce methane or formic acid (Ul = 0.58 eV) [77]. In contrast, downward polarization (P↓) stabilizes the intermediate formate (*OCHO) and ultimately tends to produce methanol (Ul = 0.64 eV). This catalytic mechanism is supported by both computational and experimental investigations, making ferroelectric materials a unique class of CO_2_RR catalysts with dynamically tunable reaction pathways.

## Research Progress of Ferro-Catalytic Materials for CO_2_RR

### Ferroelectric photocatalysts

The persistent challenge of rapid charge carrier recombination in photocatalysis has motivated innovative material design strategies. Recent advances demonstrate that ferroelectric semiconductors offer a promising solution through their intrinsic polarization-induced internal electric fields, which enable anisotropic charge separation [47–49]. This section critically examines progress in photocatalytic CO_2_RR over both traditional ferroelectric systems and emerging 2D materials (Table).

**Table.** Comparison of CO_2_RR performance of ferroelectric photocatalysts

Photocatalyst	Reaction conditions	Product	Production rate (μmol g^−1^ h^−1^)	Ferroelectric property	Reference
Bi_4_Ti_3_O_12_ nanosheets	300 W Xe-lamp	CH_3_OH	39.10	Unpolarized state	[78]
		CH_3_CH_2_OH	30.61		
Bi_2_MoO_6_ nanosheets	Solar simulator	CO	1.36	/	[92]
Polarized Bi_4_Ti_3_O_12_ nanosheets	300 W Xe-lamp	CH_3_OH	72.00	Ferroelectric polarization, *P*_max_ ≈ 0.6 μC/cm^2^	[78]
		CH_3_CH_2_OH	18.96		
SrBi_4_Ti_4_O_15_ nanosheets	300 W Xe-lamp	CH_4_	19.8	Ferroelectric polarization, *P*_max_ ≈ 1.3 μC/cm^2^	[79]
Polarized ultrathin-Bi_2_MoO_6_ nanosheets	Solar simulator	CO	14.38	Ferroelectric polarization, *P*_max_ ≈ 0.8 μC/cm^2^	[92]
V_S_-CuInP_2_S_6_ film	Solar simulator	CH_4_	82.39	Ferroelectric polarization and 2D structure	[65]
Bi_3_TiNbO_9_ nanosheets	300 W Xe-lamp	CO	20.91	Ferroelectric polarization and O_V_ engineering, *P*_max_ ≈ 0.15 μC/cm^2^	[102]
	300 W Xe-lamp	CH_4_	0.96		
Bi_0.5_Na_0.5_TiO_3_, powder	300 W Xe-lamp	CH_4_	2.28	Ferroelectric polarization and O_V_ engineering	[103]
BiFeO_3_-CdS sheet/particle nanocomposite	300 W Xe-lamp	CO	88	Ferroelectric polarization and heterojunction engineering	[141]
g-C_3_N_4_/BaTiO_3_ nanosheet/fiber	300 W Xe-lamp	CH_3_OH	18.17	Ferroelectric polarization and heterojunction engineering, *P*_max_ ≈ 16 μC/cm^2^	[121]
		CH_3_CH_2_OH	7.51		
Bi_4_Ti_3_O_12_/Au, sheet/single-atom arrays structure	300 W Xe-lamp	CO	34.15	Ferroelectric polarization and SACs engineering, *P*_max_ ≈ 0.25 μC/cm^2^	[132]

#### Traditional ferroelectric photocatalysts

Initially, investigations on ferroelectric material-based photocatalysts were mainly focused on conventional ferroelectrics, such as perovskite and Bi-based layered structures. Jia et al. [78] synthesized ferroelectric Bi_4_Ti_3_O_12_ nanosheets via molten salt method. Photocatalytic tests revealed impressive CO_2_-to-fuel conversion efficiencies, yielding CH_3_OH (39.10 μmol g^−1^ h^−1^) and CH_3_CH_2_OH (18.96 μmol g^−1^ h^−1^) as primary products. The energetically favorable CB position (−1.2 V versus normal hydrogen electrode) of Bi_4_Ti_3_O_12_ provides substantial thermodynamic impetus for CO_2_RR. Photogenerated holes predominantly migrate to the [Bi_2_O_2_] layer, while electrons primarily travel to the [TiO_6_] octahedral layer, facilitating the overall transformation process. In 2019, Tu et al. [79] developed a layered perovskite SrBi_4_Ti_4_O_15_ (SBTO) nanosheets, which exhibited ferroelectric-enhanced photocatalytic CO_2_ conversion performance. The maximum CH_4_ production rates reached 19.8 μmol h^−1^ g^−1^ (apparent quantum yield: 1.33% at 365 nm) for SBTO nanosheets with optimized ferroelectric properties, representing superior performance relative to benchmark photocatalysts. Complementary characterizations established a direct correlation between [100]-oriented spontaneous polarization and enhanced charge separation efficiency, accounting for the superior photocatalytic activity. Moreover, DFT calculations revealed that both electrons and holes show the smallest effective mass along the *a* axis, indicating high charge mobility facilitated by ferroelectric polarization. Additionally, Li et al. [80] reported that Bi_2_MoO_6_ ferroelectric nanosheets, treated by corona poling, exhibited excellent CO_2_ reduction activity for CO production, achieving a rate of 14.38 μmol g^−1^ h^−1^ in a gas–solid system without the need for sacrificial agents or co-catalysts. This efficiency represents an enhancement of more than one order of magnitude compared to bulk Bi_2_MoO_6_. The combined approach of corona poling and layered structure significantly improves the separation of photogenerated electrons and holes while increasing the reactive sites for CO_2_ adsorption, resulting in markedly enhanced photocatalytic CO_2_RR activity. These findings collectively demonstrated the emerging application of ferroelectric materials for photocatalytic CO_2_ conversion.

#### 2D ferroelectric photocatalysts

Over recent decades, 2D materials have garnered significant interest in catalytic applications, particularly as electrocatalysts and photocatalysts [81–85], due to their advantageous structural and electronic characteristics. These include high surface-to-volume ratios, abundant catalytic sites, minimized charge migration distances, and adjustable electronic band structures. Among these, piezo-/ferroelectric 2D materials (e.g., SnSe, GeS, MXenes, BiOCl, AgBiP_2_Se_6_, and In_2_Se_3_) have been extensively studied through both experimental and computational approaches for various catalytic processes [30,54,86–90], including HER, oxygen reduction reaction (ORR), NRR, and CO_2_RR. Notably, recent computational studies suggest that 2D ferroelectric heterostructures, especially those with van der Waals (vdW) interfaces, may demonstrate exceptional photocatalytic CO_2_ conversion efficiency [91], which is attributed to efficient charge carrier separation and transfer enabled by robust interlayer electronic coupling.

**Fig. 4.**
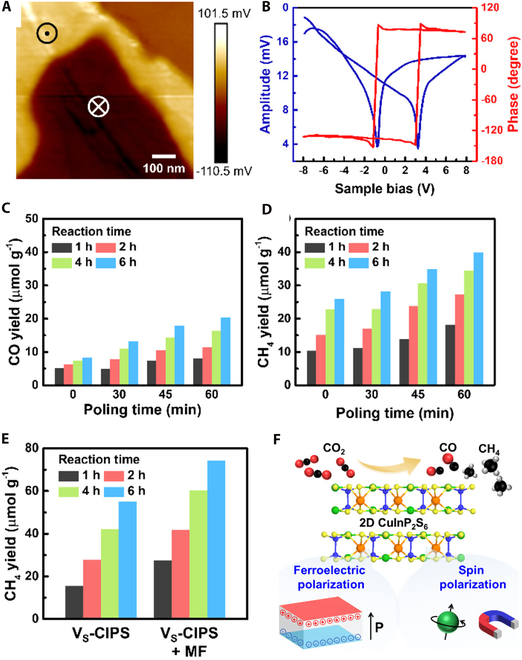
Ferroelectric and spin polarization-modulated photocatalytic CO_2_RR over 2D CuInP_2_S_6_ (CIPS). (A) OP-PFM [piezoresponse force microscopy (PFM) in the OP direction] characterization of a 200-nm-thick CIPS nanoflake. (B) Corresponding local hysteresis loops showing PFM amplitude and phase responses. (C) Temporal evolution of CO and (D) CH_4_ generation under varying poling durations (0, 30, 45, and 60 min). (E) Comparative analysis of CH_4_ yield between V_S_-CIPS and magnetic field-assisted V_S_-CIPS systems at different reaction time intervals (1, 2, 4, and 6 h). (F) Schematic illustration of the mechanism of ferroelectric and spin polarization promoted catalysis in 2D CIPS. Reproduced with permission from [65]. Copyright 2024, American Chemical Society.

Recent work by Chiang et al. [65] has revealed that simultaneous control of ferroelectric and spin polarizations in 2D CuInP_2_S_6_ (CIPS) can significantly enhance photocatalytic CO_2_ reduction performance (Fig. 4A and B). Controlling the ferroelectric–paraelectric phase transition and applying electrical poling treatments lead to increased CO and CH_4_ production with extended polarization time. Optimal product yields of 20.34 μmol g^−1^ (CO) and 39.88 μmol g^−1^ (CH_4_) were achieved after 60 min of polarization during 6-h catalytic tests (Fig. 4C and D). Further performance enhancement was realized through synergistic sulfur vacancy (V_S_) engineering and magnetic field application, where the spin-polarized V_S_-CIPS system demonstrated substantially improved activity relative to pristine CIPS. The modified V_S_-CIPS system exhibited a photocatalytic CH_4_ yield of 54.9 μmol g^−1^, which increased by 35% to 74.29 μmol g^−1^ under magnetic field (Fig. 4E and F). These findings establish a new strategy for enhancing CO_2_ photoreduction in 2D ferroelectric materials through combined defect engineering and external field control. Similarly, intrinsic polarization-induced surface band edge modifications and efficient photocatalytic CO_2_RR were also observed in other 2D ferroelectric systems like CuBiP_2_Se_6_ [92].

Moreover, Li and Young [93] conducted DFT calculations to study the photocatalytic CO_2_RR activity of a novel 2D MXene Y_2_CO_2_ ferroelectric material with a suitable bandgap. The material’s capability to convert CO_2_ into various C_1_ products, including CO, formic acid, methanol, and methane, was systematically investigated. The results revealed that on an oxygen-vacancy (O_V_) defective Y_2_CO_2_ surface, CO_2_ adsorption is strongly preferred over CO adsorption on the “poled up” surface (*E*_ads_ = −2.27 eV for CO_2_ versus −2.12 eV for CO), while this preference reverses on the “poled down” surface (*E*_ads_ = −1.56 eV for CO_2_ versus −2.87 eV for CO). This polarization-dependent adsorption behavior enables selective molecular adsorption control. Furthermore, the study found that methanol formation is more favorable on the “poled up” surface (*E*_ads_ = −0.76 eV for CH_3_OH) compared to the “poled down” surface (*E*_ads_ = −1.14 eV), while formic acid shows stronger adsorption on the “poled down” surface (*E*_ads_ = −1.40 eV versus −0.93 eV on “poled up”). Notably, methane adsorption remains weak on both surfaces (*E*_ads_ > −0.5 eV), making CH_4_ formation energetically unfavorable. This work demonstrates that 2D Y_2_CO_2_ MXene, with its tunable adsorption properties through polarization control, represents a highly promising and efficient catalyst for CO_2_RR, providing valuable guidance for designing advanced 2D ferroelectric photocatalysts.

### Engineering strategies of ferroelectric photocatalysts

For photocatalytic process, increasing the polarization field of ferroelectric catalysts can effectively modulate the surface electronic state and enhance the separation efficiency of photogenerated electron–hole pairs, offering significant potential to greatly improve the activity and selectivity of photocatalytic reactions. Consequently, the current engineering strategies for ferroelectric photocatalysts mainly focus on enhancing the polarization field, optimizing the adsorption modes and increasing surface active sites [94,95]. These strategies include engineering oxygen vacancy, constructing heterojunction, and introducing single-atom catalysts (SACs). The following section provides a comprehensive overview of the fundamental principles underlying these promotion strategies, along with the most illustrative examples reported recently.

#### Engineering oxygen vacancy

Oxygen vacancies (O_V_) appear in oxygen-containing compounds because of the lack of oxygen atoms in the crystal lattice [96]. The O_V_, acting as donor-like defects, can introduce a defect band near the CB of the metal oxide photocatalyst [97]. The presence of oxygen vacancies and the resulting saturation of metal coordination contribute to CO_2_ capture and electron generation reactions, ultimately promoting the CO_2_ photocatalytic reduction process [98]. The O_V_ in the catalyst serves as an effective adsorption site for CO_2_ molecules. The insertion of an oxygen atom from the CO_2_ molecule into the O_V_ leads to the formation of a stable adsorption complex. During the CO_2_ adsorption process, the *d*-orbital of the coordinated unsaturated metal atom contributes additional electrons to the lowest unoccupied molecular orbital (LUMO) of the CO_2_ molecule [99]. This electron transfer process not only increases the electron density of the CO_2_ molecule but also facilitates the breaking of chemical bonds. In particular, the electron transfer induces a distortion of the CO_2_ molecule’s structure, thereby reducing its chemical bonding energy and increasing its susceptibility to subsequent reaction steps [100].

**Fig. 5.**
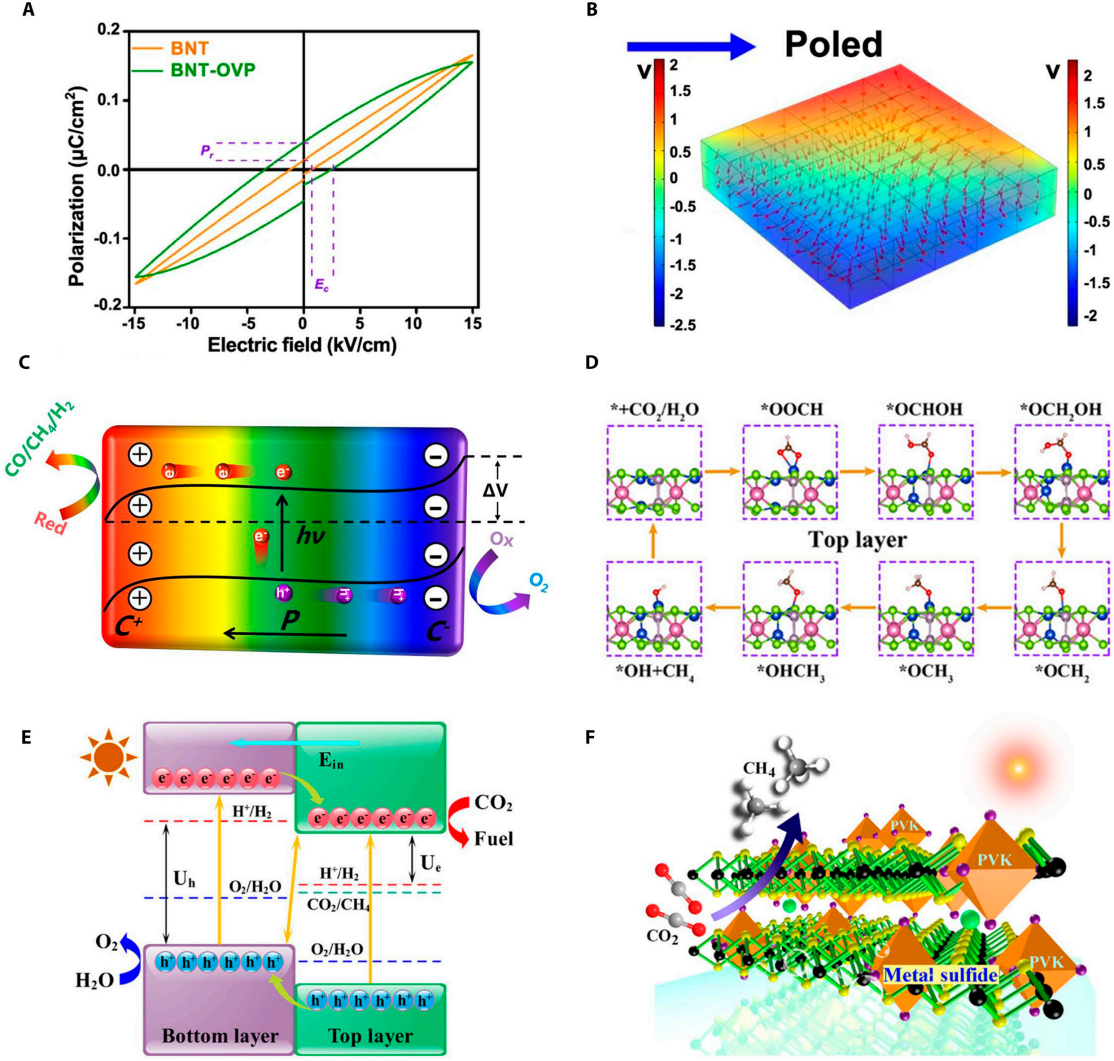
(A) Ferroelectric hysteresis characteristics of pristine Bi_3_NbTiO_9_ (BNT) versus oxygen vacancy-engineered BNT-OVP. (B) Finite element modeling of the poling-generated internal electric field distribution in polarized BNT nanosheets. (C) Schematic illustration of the ferroelectric catalysis mechanism and tilting of energy bands under polarization field and the accompanied redox reactions. Reproduced with permission from [102]. Copyright 2021 Springer Nature. (D) Proposed reaction routes for CO_2_RR. (E) Schematic diagram of the 2D ferroelectric bilayer for CO_2_RR and OER. Reproduced with permission from [91]. Copyright 2021 American Chemical Society. (F) Conceptual illustration of ferroelectric–semiconductor hybrid systems employing metal sulfides for photocatalytic CO_2_ conversion. Reproduced with permission from [120]. Copyright 2019 American Chemical Society.

Incorporating oxygen vacancies into ferroelectric materials not only enhances their ability to absorb visible light (by shifting the CB downward) but also significantly alters the materials’ charge transfer characteristics [101]. As an active site, O_V_ plays a crucial role in modulating the photocatalytic CO_2_ reduction processes. A represetative study by Zhang and colleagues [102] reported ferroelectric Bi_3_NbTiO_9_ (BNT) nanosheet catalysts prepared using a one-pot hydrothermal method by creating surface oxygen vacancies through corona polarization treatment. The *P–E* hysteresis loop reveals that corona poling treatment and O_V_ engineering synergistically enhance the built-in electric field polarization (Fig. 5A). Subsequent calculations reveal that the carrier density values for BNT, BNT-P, BNT-OV2, and BNT-OVP are 3.46 × 10^21^, 4.44 × 10^21^, 3.89 × 10^21^, and 5.63 × 10^21^, respectively. The flat-band potential remains unaffected by corona polarization and/or the formation of O_V_. These results demonstrate that the improved charge separation efficiency of BNT-OVP stems from a synergistic combination of elevated ferroelectric spontaneous polarization and the presence of O_V_. In addition, COMSOL simulations indicate that the incorporation of O_V_ hinders the reverse switching of domains in the BNT nanoplates, resulting in a sustained elevated residual polarization-induced electric field (Fig. 5B). The positively polarized charge facilitates the reduction reaction, while the negatively polarized charge promotes oxidation reactions (Fig. 5C). This results in an enhanced photocatalytic CO_2_RR activity of the BNT nanosheets. The presence of O_V_ creates a “pin effect” on the switching of domain poles, which in turn affects the separation of electrons and holes. Additionally, the incorporation of O_V_ increases the CO_2_ adsorption energy from 0.990 to 1.808 eV, supporting the idea that O_V_ facilitates the CO_2_ adsorption and reduction process.

A very recent work by Liu et al. [103] demonstrated that introducing O_V_ in ferroelectric Bi_0.5_Na_0.5_TiO_3_ can create a defect level, which in turn effectively modulates the band structure, reduces the barrier for carrier transport, and promotes the separation of photogenerated electron–hole pairs. Specifically, O_V_ engineering alters the local chemical environment of Bi_0.5_Na_0.5_TiO_3_, enhancing the intensity of spontaneous polarization through lattice distortion. Compared to other ferroelectric photocatalysts, Bi_0.5_Na_0.5_TiO_3_ with O_V_ defects significantly enhances CO yield and product selectivity. Similarly, this strategy for constructing O_V_ can also be applied to other polar materials systems, such as BiOIO_3_ nanoribbon [104], allowing it to exhibit excellent CO_2_ photoreduction properties that surpass those of most reported gas–solid reaction catalytic systems.

#### Selective loading of co-catalyst

Anchoring co-catalysts represents a promising approach to improve the performance of photocatalytic materials [105]. Ferroelectric materials can direct the selective deposition of redox co-catalysts on different polar facet. The inherent spontaneous polarization of ferroelectric materials creates strong internal electric fields that drive the spatial separation of photogenerated charge carriers, accumulating electrons on one polar facet and holes on the opposite facet [53]. This charge separation provides a powerful driving force for the selective anchoring of reduction co-catalysts (e.g., Pt or Ag) onto the electron-rich facet and oxidation co-catalysts (TiO_2_, MnO*_x_*, or Co_3_O_4_) onto the hole-rich facet [106,107], thereby dramatically enhancing photocatalytic efficiency by minimizing charge recombination, providing dedicated active sites for each half-reaction, spatially separating incompatible products to prevent back-reactions, and improving overall reaction kinetics and stability.

Yang et al. [108] employed photodeposition method to load AuCu and MnO*_x_* cocatalysts on oppositely poled facets of ferroelectric PbTiO_3_ nanoplates. A band bending occurs along with the formation of built-in electric fields near the semiconductor–cocatalyst interfaces, together with an intrinsic ferroelectric polarization field, which provide a strong driving force for the directional drift of photogenerated electrons and holes toward AuCu and MnO*_x_*, respectively, resulting in significantly enhanced photocatalytic activity.

#### Constructing ferroelectric–semiconductor heterojunctions

Heterojunctions represent interfacial regions formed by the junction of 2 dissimilar semiconductors exhibiting distinct electronic band configurations, thereby generating built-in electric fields that improve charge carrier separation and transport under illumination [109–113]. Utilizing the electric field generated in ferroelectric materials and heterojunctions to synergistically enhance the separation of electrons and holes is considered a highly promising approach for developing efficient photocatalysts [114,115]. Incorporating ferroelectric materials can regulate the degree of band bending through manipulation of spontaneous polarization and enhance the strength of the built-in electric field within the heterojunction [116].

Heterojunction photocatalysts are classified into 3 main categories: type II, type S, and type Z heterojunctions [59,117–119]. Type II heterostructures comprise 2 semiconductor materials with staggered band alignment. This energetically favorable configuration induces spontaneous spatial separation of photogenerated charge carriers, yielding enhanced photocatalytic activity beyond the capabilities of individual semiconductor components [98,115]. Implementation of this design principle in 2D CIPS bilayers demonstrated that the intrinsic polarization field effectively suppresses charge recombination with predominant CH_4_ production (Fig. 5D and E) [91]. Notably, a high solar-to-fuel efficiency of 8.40% was achieved over the CIPS bilayer heterojunctions. An in situ atom-sharing strategy has been used to prepare Cs_2_SnI_6_/SnS_2_ heterojunction. Compared to pure ferroelectric SnS_2_, the photocatalytic CO_2_RR performance of the heterojunction was significantly enhanced by about 5 times (Fig. 5F) [120]. This strategy can be extended to the preparation of various perovskite–metal dihalide heterostructures (Cs_3_M_2_X_9_/M_2_Y_3_, where M = Bi, Sb; X = Br, I; Y = S, Se). Similarly, well-constructed g-C_3_N_4_/BaTiO_3_ heterojunction photocatalyst also showed significantly enhanced reverse movement of photoexcited carriers due to the coupling effect of the heterojunction and ferroelectric polarization, effectively reducing the recombination and substantially improving the CO_2_RR performance [121]. The classical Z-scheme configuration integrates 2 semiconductor photocatalysts with staggered band alignment coupled with an oxidation–reduction mediator system. Z-type BiFeO_3_/CsPbBr_3_ heterojunction has been demonstrated and resulted in a notable enhancement in catalytic activity [122]. Type S heterostructures are constructed by combining a reducing-type semiconductor photocatalyst with a smaller work function and higher Fermi level, and an oxidizing-type semiconductor photocatalyst with a larger work function and lower Fermi level in an offset configuration [123]. Well-designed 2D/2D S-scheme heterojunction composed of ferroelectric Bi_3_TiNbO_9_ and C_3_N_5_ nanosheets was reported, achieving efficient charge transfer through the depolarization field [124]. Further studies are needed to extend their application to CO_2_ reduction and reveal the role of ferroelectric polarization in these composite catalysts.

**Fig. 6.**
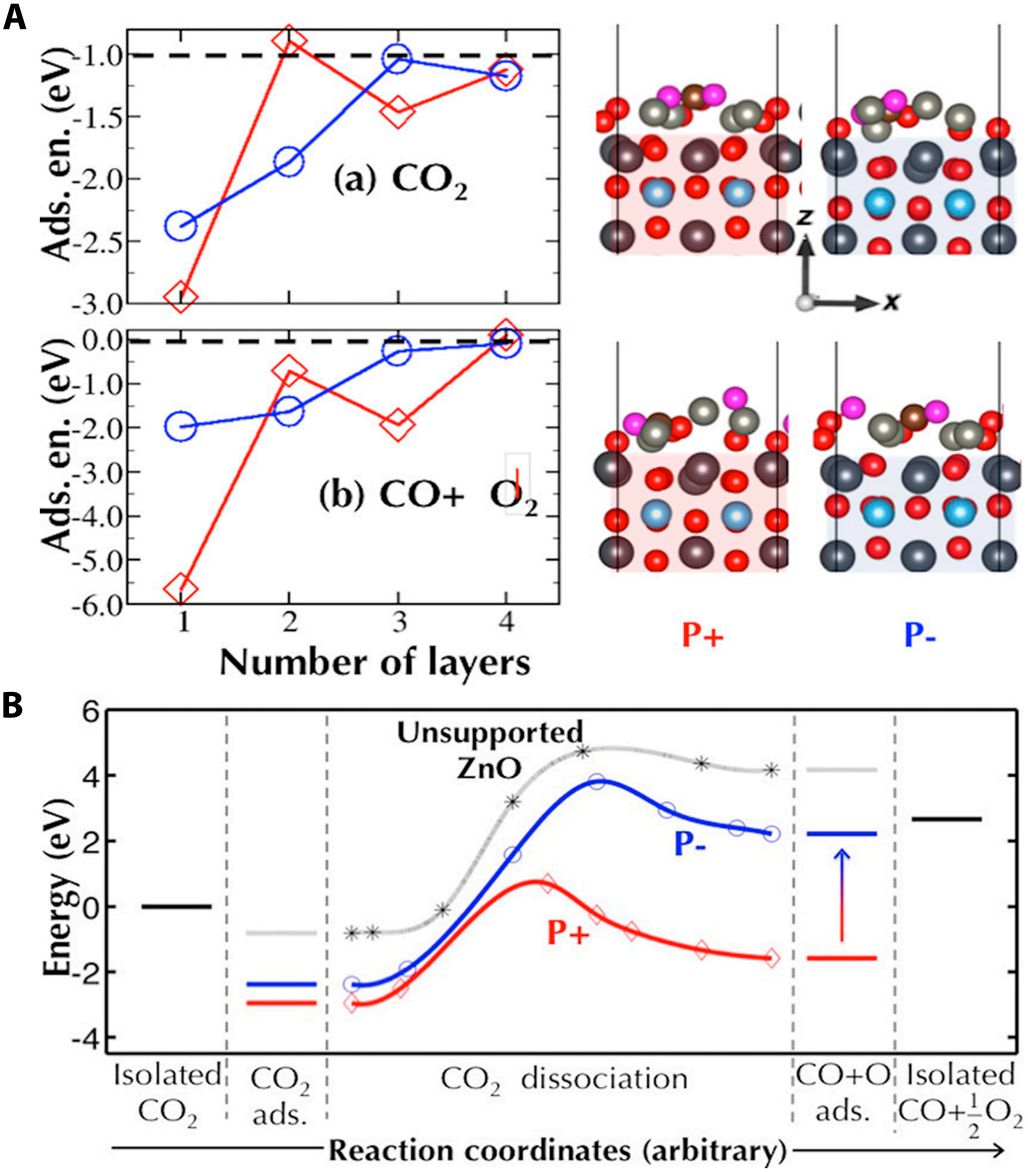
(A) Dependence of CO_2_ and CO + O_2_ adsorption energies on (ZnO(112̅0))*_n_*/[2 × 2]PbTiO_3_ as a function of layer number (*n*) for both positive and negative polarization states. Corresponding molecular adsorption geometries on the stoichiometric (*n* = 1) configuration are displayed on the right. (B) Comparative analysis of CO_2_ dissociation pathways on pristine ZnO versus polarization-dependent PbTiO_3_-supported ZnO catalysts. Reproduced with permission from [125]. Copyright 2016, American Chemical Society.

Besides experimental work, theoretical simulations have also been conducted on ferroelectric–semiconductor heterostructures. For example, Alawode and Kolpak [125] introduced an innovative dynamically adjustable stoichiometric ZnO(112̅0)*_n_*/PbTiO_3_ catalytic system, where surface reactivity is modulated by the PbTiO_3_ substrate’s polarization orientation and the ZnO(112̅0) layer thickness (Fig. 6). This configuration results in a substantially reduced energy pathway for CO_2_ transformation. Their findings revealed pronounced variations in CO_2_ adsorption energy upon polarization reversal, implying that reaction thermodynamics and reaction routes for CO_2_ activation can be precisely regulated, as evidenced by the dynamic polarization-switching mechanism proposed for CO_2_ dissociation on ZnO(112̅0)/PbTiO_3_. Such a strategy may extend to more heterogeneous structures, offering new opportunities for manipulating reaction energetics.

#### Single-atom ferroelectric nanohybrids

The concept of SACs was first reported by Qiao et al. [126] as a novel catalyst type consisting of dispersed single metal atoms as the active centers [127,128]. The monatomic dispersion of SACs allows each isolated metal atom to participate in catalytic reactions, significantly enhancing the efficiency of chemical processes. In the CO_2_RR, the highly stable molecular structure of CO_2_ leads to a higher dissociation energy for the C=O bond compared to other chemical bonds, such as C–C or C–H bonds [129]. This indicates that the reduction of CO_2_ requires a higher activation energy. Concurrently, the dissociation of the C=O bond requires the involvement of multiple electron and proton transfer processes, as well as complex multi-step reactions [130]. This makes enhancing reaction selectivity challenging. In this context, SACs utilize the unsaturated *d*-orbitals of transition metals (TMs) for electron transfer with CO_2_ molecules, facilitating the formation of a more reducible intermediate and lowering the dissociation energy of the C=O bond [92]. In this sense, ferroelectric substrates provide ideal platforms for stabilizing isolated metal atoms, creating ferroelectric-supported SACs [92,131]. Such hybrid systems synergistically integrate the switchable polarization of ferroelectrics with the exceptional reactivity of atomic metal sites, leading to enhanced performance in CO_2_RR with regard to both activity and product selectivity.

**Fig. 7.**
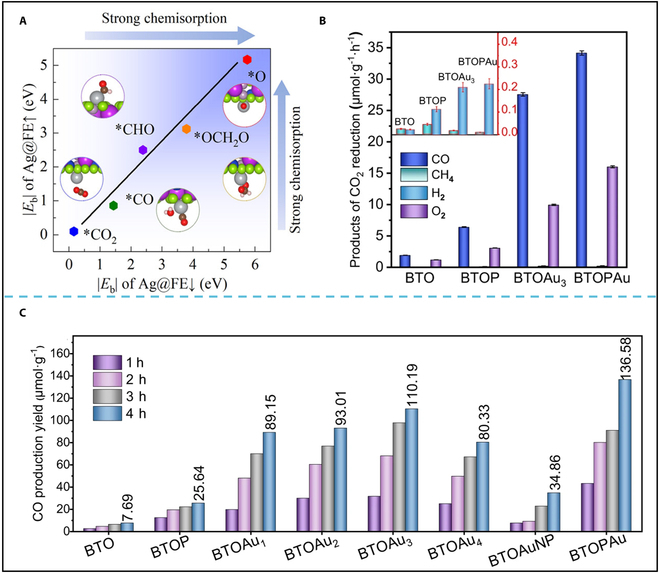
(A) Conceptual illustration of polarization-modulated CO_2_ reduction pathways. Reproduced with permission from [92]. Copyright 2024 American Chemical Society. (B) Evolution rates for CO, CH_4_, H_2_, and O_2_ over BTO, poled BTOP, BTO with varying Au cluster sizes, Au nanoparticle-loaded BTO (BTOAuNP), and poled BTO with atomic Au (BTOPAu). (C) Photocatalytic CO generation efficiency in a gas–solid phase system under simulated solar light. Reproduced with permission from [132]. Copyright 2024 Springer Nature.

Jiang et al. [92] performed DFT simulations to elucidate the CO_2_RR mechanism in Ag@CuBiP_2_Se_6_ system, where ferroelectric polarization cooperates with single-atom catalytic sites (Fig. 7A). Computational results reveal that the ferroelectric CuBiP_2_Se_6_ matrix generates a built-in polarization field, which drives directional charge carrier separation and transport. Meanwhile, Ag SAC anchored on the substrate can serve as an electron reservoir to modify the bonding configuration of the surface through appropriate static electron transfer, effectively promoting the adsorption and activation of CO_2_ molecules. The complementary effects between the ferroelectric-induced bulk polarization and atomic-level charge regulation at Ag sites collectively enhance CO_2_ capture and conversion efficiency. Notably, switching the ferroelectric polarization can selectively produce 2 different products (CH_4_ or CH_3_OH), providing further evidence of the feasibility of regulating the reaction pathway through a ferroelectric polarization switch.

In addition, Liu et al. [132] reported the modulation of periodic 1D Au single-atom arrays by polarization electric field of ferroelectric Bi_4_Ti_3_O_12_ nanosheets. The Au single-atom arrays significantly reduce the Gibbs free energy for CO_2_RR through Au-O=C=O-Au dual-site adsorption compared to that on Au isolated single atoms. Moreover, the Au single-atom arrays hinder the depolarization of Bi_4_Ti_3_O_12_, maintaining a stronger driving force for separating photogenerated charges. As a result, Bi_4_Ti_3_O_12_ with Au single-atom arrays displays a high CO production rate of 34.15 μmol·g^−1^·h^−1^, which is 18 times higher than that of pristine Bi_4_Ti_3_O_12_ (Fig. 7B and C). More importantly, the polarization electric field proves to be a general tactic for the synthesis of 1D Pt, Ag, Fe, Co, and Ni single-atom arrays on the ferroelectric Bi_4_Ti_3_O_12_ surface. The experimental and computational evidence collectively demonstrates that engineering ferroelectric-supported SACs establishes a dual-functional platform for optimizing both reaction kinetics and product distribution of photocatalytic CO_2_ reduction.

### Ferroelectric electrocatalysts

Besides intensively investigated photocatalysis, ferroelectric materials have also been applied to the field of electrocatalysis recently. In electrocatalysis, the switchable ferroelectric polarization effect can directly control gas adsorption strength, overpotential, and selectivities. In addition, it can also regulate the orbitals of the surface metal atoms. Consequently, this effect can not only activate the reactants effectively but also play a crucial role in enhancing the activity and selectivity of electrocatalytic CO_2_RR [77].

**Fig. 8.**
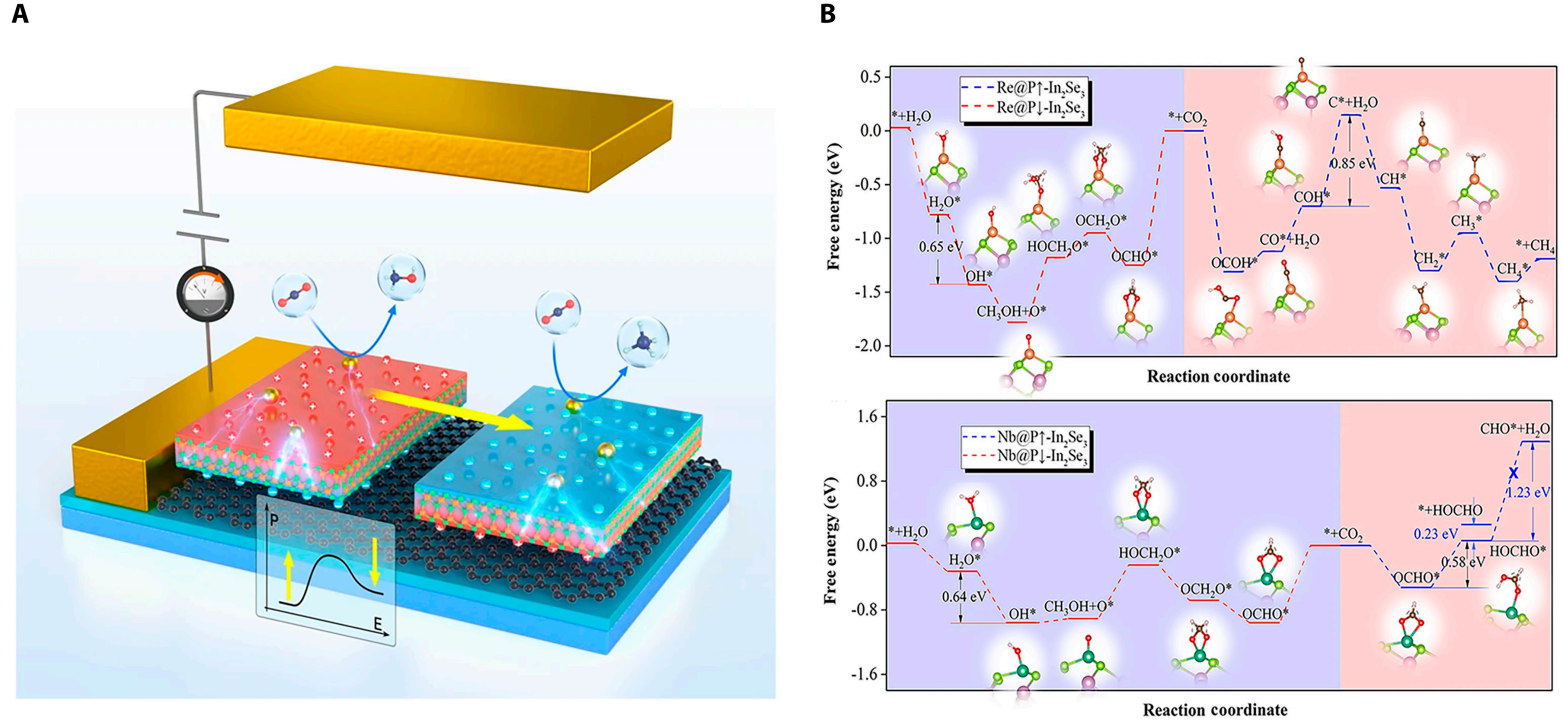
(A) CO_2_RR pathways on Re@In_2_Se_3_ and Nb@In_2_Se_3_. (B) Adsorption energy of the intermediate on Ag@FE↑ versus Ag@FE↓ surface. Reproduced with permission from [77]. Copyright 2021 Springer Nature.

The single-atom engineering strategy has also been demonstrated to be applicable to the field of electrocatalysis [133]. The switchable electric polarization in layered In_2_Se_3_ can dynamically tune the activation barriers, intermediate stabilization, and selectivity in electrocatalytic CO_2_RR catalyzed by isolated TM atoms (Fig. 8A). Based on the single-atom ferroelectric nanohybrid construction strategy, 6 TMs, including Ni, Nb, Pd, Re, Rh, and Zr, were identified as potential catalysts for electrochemical CO_2_RR using DFT calculations [77]. These findings show that the inversion of polarization in single-layer ferroelectric In_2_Se_3_ can regulate the energies of empty and occupied *d*-orbitals, as well as *d*-electrons of adsorbed TM atoms, thereby optimizing catalytic performance in terms of CO_2_ activation, limiting potential, reaction pathway, and final product composition. Notably, the Re@In_2_Se_3_ and Nb@In_2_Se_3_ catalysts can selectively produce different desired products on the same catalyst through ferroelectric polarization switching (Fig. 8B). Compared to conventional nonferroelectric substrates, such as Pd@C_3_N_4_, TMs exhibit significantly lower limit potentials and unique ferroelectric-controllable CO_2_ catalytic properties using In_2_Se_3_. Additionally, computational investigations employing reversible hydrogen electrode (RHE) and fixed-potential methodologies were conducted on TM single atoms (M = Fe, Co, Ni, Cu) supported on monolayer α-In_2_Se_3_, systematically evaluating polarization-switching effects on CO_2_ electroreduction mechanisms [134]. The results indicate thermodynamic preference for *OCHO intermediate formation during CO_2_ protonation, establishing this pathway as the predominant initiation step across the M@In_2_Se_3_ series. The computational screening identifies Co-embedded In_2_Se_3_ with carbon coordination (Co@In_2_Se_3_-C) as the optimal candidate, exhibiting the lowest theoretical overpotential (−0.385 V versus RHE) among the evaluated systems.

In addition, the synergistic effect between the metal cluster and ferroelectric substrate is anticipated to impact the CO_2_RR performance substantially. Yang et al. [135] comprehensively examined CO_2_ electroreduction pathways on Cu_3_ trimer catalysts supported by polar Janus MoSX (X = Se, Te) monolayers using DFT calculations. The broken vertical symmetry in these Janus structures induces intrinsic out-of-plane dipoles, creating asymmetric interfacial environments for Cu_3_ cluster stabilization on opposing polar facets. The computational results establish a strong correlation between substrate polarization vectors (both magnitude and orientation) and the CO_2_ reduction activity of supported Cu_3_ ensembles. Among all configurations, the S-terminated MoSTe-supported Cu_3_ catalyst [Cu_3_(S)@MoSTe] achieved peak performance for CH_4_ generation, exhibiting 3.2-fold higher activity than Se-terminated counterparts. This work elucidates the pivotal influence of Janus substrate polarization on adsorbate binding energetics, where tailored interfacial electric fields optimize intermediate stabilization and overall catalytic efficiency.

## Conclusion and Perspective

**Fig. 9.**
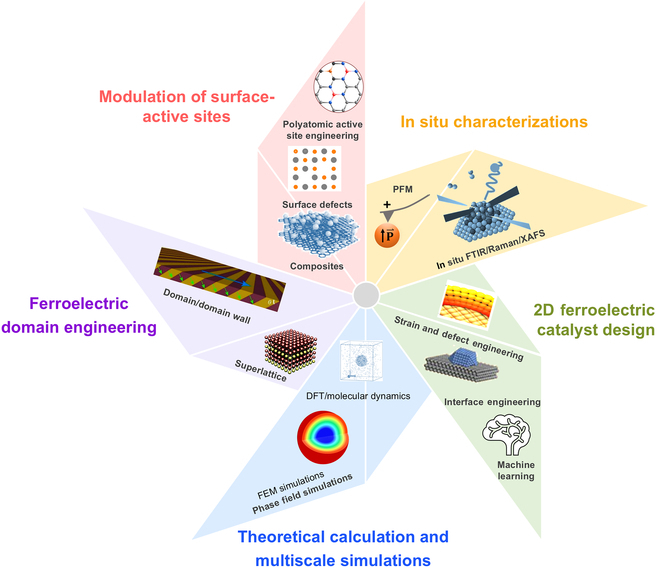
Schematic illustration of future research directions in photo-/electrocatalytic CO_2_RR using ferroelectric materials.

Ferroelectric materials possess spontaneous polarization and reversible switching properties that enable precise modulation of catalytic activity and selectivity in CO_2_ reduction for both photocatalytic and electrocatalytic pathways. These effects can significantaly tune the surface charges, electronic structure, adsorption behavior of reactants and products, and separation and migration of free charges, leading to controllable catalytic reaction pathways and enhanced photo-/electrocatalytic CO_2_RR selectivity and efficiency. In this short review, we comprehensively summarized the reaction mechanisms of ferroelectric polarization-modulated CO_2_RR, highlighted key advances in ferroelectrics-based photocatalyts/electrocatalysts for CO_2_RR, and discussed new strategies to enhance the catalytic efficiency of ferroelectric materials. While significant progress has been made in the field of ferroelectric catalysis for CO_2_RR, there are still many challenges to be addressed. Future research should focus on the design of novel high-efficient ferroelectric catalytic materials, the deeper understanding of the underlying mechanisms, and the development of synergistic strategies to achieve more efficient and selective CO_2_ reduction (Fig. 9).

First, future research should prioritize the rational design and well-controlled synthesis of high-efficient ferroelectric photo/electrocatalysts. Modulation strategies of ferroelectric catalysts include creating surface defects, introducing active sites, and tuning ferroelectric domain/domain walls. In particular, the emerging 2D ferroelectrics are the most promising candidates (e.g., SnSe [136,137], GaSe, Bi_2_O_2_Se [138], and CIPS [65]), which can be well controlled by modulating the strain/defects to enhance the spontaneous polarization. The key industrial challenges of 2D ferroelectric catalysts include their high production costs and stability. Therefore, high-throughput screening is required to combine with machine learning algorithms to build a database of 2D ferroelectric materials (including descriptors such as band gap, polarization strength, and adsorption energy) and screen for stable and low-cost ferroelectric materials and optimal ferroelectric–co-catalyst combinations. Moreover, polarization stability degradation due to the screening effect should also be carefully addressed, which can be improved by forming a ferroelectric–semiconductor heterojunction [139].

Second, a deeper understanding of the fundamental mechanisms underlying ferroelectric photo-/electrocatalysis is essential for material design and optimization of selectivity toward desired CO_2_RR products. Research should focus on identifying the key intermediates and transition states involved in these pathways and how they are influenced by the polarization field. In situ characterization techniques are desiable (e.g., electrochemical–piezoelectric force microscopy system) for directly detecting the evolution of the surface charge distribution during polarization flip in real time. Moreover, in situ spectroscopic techniques including surface-enhanced Raman spectroscopy (TR-SERS) and Fourier transform infrared (FTIR) can be used to reveal the dynamic changes of key intermediates (*COOH, *CO, etc.). Besides, multiscale simulations such as first-principles calculations and molecular dynamics simulations are required to reveal the effect of polarization direction on the energy barrier and the reaction kinetics, and finite element method (e.g., COMSOL) can be employed to simulate the electric field distributions.

Third, synergistic strategies combining multiple effects should be explored to combine ferroelectric polarization with complementary effects to improve CO_2_RR performance. The optimization of ferroelectric catalysts is chiefly concerned with enhancing polarization and adsorption capacity. Vacancy engineering, heterojunctions, and SACs have been identified as effective strategies for enhancing CO_2_RR activity. Consequently, further exploration is necessary to integrate diverse strategies to optimize the synergistic effect. For instance, for ferroelectric–photothermal synergy, the localized heating risks depolarization above *T*_c_, and thus, alternative approaches (e.g., plasmonic heating below *T*_c_, or strain-engineered thermally stable ferroelectrics) should be explored. Also, polyatomic active site engineering, which utilizes stabilizing complementary intermediates on the polarized surfaces (e.g., CO_2_-activated and C–C-coupled sites), is promising for selectively form multicarbons. Moreover, spatially graded reactors need to be developed, which can tailor polarization gradients to guide reactants through optimized adsorption, activation, and desorption zones.
